# Identification of trans-genus biomarkers for early diagnosis of intestinal schistosomiasis and progression of gut pathology in a mouse model using metabolomics

**DOI:** 10.1371/journal.pntd.0011966

**Published:** 2024-02-21

**Authors:** Peerut Chienwichai, Phornpimon Tipthara, Joel Tarning, Yanin Limpanont, Phiraphol Chusongsang, Yupa Chusongsang, Nuttapohn Kiangkoo, Poom Adisakwattana, Onrapak Reamtong

**Affiliations:** 1 Princess Srisavangavadhana College of Medicine, Chulabhorn Royal Academy, Bangkok, Thailand; 2 Mahidol Oxford Tropical Medicine Research Unit, Faculty of Tropical Medicine, Mahidol University, Bangkok, Thailand; 3 Centre for Tropical Medicine and Global Health, Nuffield Department of Clinical Medicine, University of Oxford, Oxford, United Kingdom; 4 Department of Social and Environmental Medicine, Faculty of Tropical Medicine, Mahidol University, Bangkok, Thailand; 5 Department of Helminthology, Faculty of Tropical Medicine, Mahidol University, Bangkok, Thailand; 6 Department of Molecular Tropical Medicine and Genetics, Faculty of Tropical Medicine, Mahidol University, Bangkok, Thailand; Aberystwyth University - Penglais Campus: Aberystwyth University, UNITED KINGDOM

## Abstract

Schistosomiasis is one of the most devastating human diseases worldwide. The disease is caused by six species of *Schistosoma* blood fluke; five of which cause intestinal granulomatous inflammation and bleeding. The current diagnostic method is inaccurate and delayed, hence, biomarker identification using metabolomics has been applied. However, previous studies only investigated infection caused by one *Schistosoma* spp., leaving a gap in the use of biomarkers for other species. No study focused on understanding the progression of intestinal disease. Therefore, we aimed to identify early gut biomarkers of infection with three *Schistosoma* spp. and progression of intestinal pathology. We infected 3 groups of mice, 3 mice each, with *Schistosoma mansoni*, *Schistosoma japonicum* or *Schistosoma mekongi* and collected their feces before and 1, 2, 4 and 8 weeks after infection. Metabolites in feces were extracted and identified using mass spectrometer-based metabolomics. Metabolites were annotated and analyzed with XCMS bioinformatics tool and Metaboanalyst platform. From >36,000 features in all conditions, multivariate analysis found a distinct pattern at each time point for all species. Pathway analysis reported alteration of several lipid metabolism pathways as infection progressed. Disturbance of the glycosaminoglycan degradation pathway was found with the presence of parasite eggs, indicating involvement of this pathway in disease progression. Biomarkers were discovered using a combination of variable importance for projection score cut-off and receiver operating characteristic curve analysis. Five molecules met our criteria and were present in all three species: 25-hydroxyvitamin D2, 1α-hydroxy-2β-(3-hydroxypropoxy) vitamin D3, Ganoderic acid Md, unidentified feature with m/z 455.3483, and unidentified feature with m/z 456.3516. These molecules were proposed as trans-genus biomarkers of early schistosomiasis. Our findings provide evidence for disease progression in intestinal schistosomiasis and potential biomarkers, which could be beneficial for early detection of this disease.

## Author summary

Schistosomiasis is a parasitic disease caused by the blood fluke *Schistosoma*. Five species of *Schistosoma* cause intestinal damage and inflammation, but the current method of diagnosis is not satisfactory because it lacks sensitivity and timeliness. Therefore, we applied metabolomics to identify the overall types and abundance of compounds in biological samples to discover new markers. Previous metabolomic studies focused only on infection with one species, making it difficult to detect disease caused by other species. Moreover, no study examined progression of intestinal disease. In this study, we used a mouse model and metabolomics to investigate changes in the gut environment from the early stage of the disease and identify markers of early infection caused by three different species of *Schistosoma*. We found that changes in compounds related to lipid metabolism since early infection and some pathways, such as glycosaminoglycan degradation, might be related to intestinal disease. We identified five compounds as early biomarkers of schistosomiasis, which were shared among the three species. These compounds could be used for future biomarker development, which could facilitate diagnosis of schistosomiasis.

## Introduction

Schistosomiasis is one of the most threatening infectious diseases that cause public health problems in many countries in Africa, Central and South America, and Asia. Approximately 800 million people, especially children, are at risk for getting schistosomiasis, leading to 250 million infections worldwide [1]. Prevalence of this disease varies from region to region, for example, 90.6% in north-western Tanzania [2], 40% in south-western Nigeria [3], 37.38% in Jiangsu Province, China [4], 8.4% in The Philippines [5] 4.9–8.1% in north-eastern Brazil [6], and 0.6% in Puerto Rico [7]. The burden of *Schistosoma* infection is substantial, including 1,430,000 disability adjusted life year (DALY) per year globally and 280,000 deaths in Sub Saharan Africa region annually [1]. Schistosomiasis can be divided into 2 forms, urogenital form, caused by *S*. *haematobium*, and intestinal form, caused by *S*. *mansoni*, *S*. *japonicum*, *S*. *mekongi*, *S*. *intercalatum*, and *S*. *guineensis*. Regarding urogenital schistosomiasis, patients show cystitis, dysuria, hematuria due to granuloma formation in urinary bladder, which severe cases may develop hydronephrosis, and squamous cell carcinoma of the urinary bladder. Likewise, intestinal schistosomiasis patients show abdominal pain and discomfort, loss of appetite, and bloody diarrhea. Some patients experience intestinal and liver symptoms, such as granulomatous inflammation and bleeding from the intestine, pseudopolyposis, liver abscess, periportal fibrosis, and eventually death [8].

Schistosomiasis patients are infected by contacting infective juvenile stage, cercariae, in freshwater. Upon contact, the juvenile *Schistosoma* parasite penetrates patient skin and transforms into schistosomula stage. The schistosomula enters blood circulation, then develops into adults. Adult worms migrate to their desired blood vessels, mate, and produce eggs, which usually take 5 weeks after infection [8]. Eggs of *Schistosoma* spp. are the main pathogenic agent of schistosomiasis because they induce inflammation and granuloma in many organs and lead to clinical symptoms of the disease [1,8]. The current gold standard for *Schistosoma* spp. detection is the parasitological examination of parasite’s eggs from patient samples, urine for urinary schistosomiasis and feces for intestinal schistosomiasis. However, the current method has a substantial drawback, the low sensitivity. It has been estimated that the sensitivity of parasitological detection is less than 50%, and its detection capacity decreases for people living in low-prevalence areas [8–9]. Many alternatives have been proposed to overcome this problem of classical methods, for example, detection of parasite’s genetic material and proteins in patient blood, but no method achieve the satisfactory results. Detection of parasite proteins, including circulating anodic and cathodic antigens (CAA and CCA), is the most promising alternative so far; however, false positives and high costs remain problematic [10–11]. Detection of cell-free parasitic DNA has gained attention lately but applying to routine field practice is still challenging [12–13]. Therefore, developing new approaches for the diagnosis of schistosomiasis is both a significant gap and a new possibility to reduce illnesses and deaths caused by this parasite.

Recently, metabolite-based diagnostic methods have been highlighted as a successful approach for point-of-care diagnosis of infectious diseases. For example, Slade, *et al*. developed a real-time volatile metabolite detection system for differentiating species of bacteria from wounds. Their findings showed that volatile metabolites were excellent markers for diagnosis of septic wounds [14]. Specifically for parasitic infections, Shirey *et al*. developed a lateral flow immunoassay targeting N-acetyl-tyramine-O-glucuronide (NATOG), a metabolite biomarker for onchocerciasis. This lateral flow assay achieved an 85% sensitivity and could distinguish current infections from past ones, surpassing basic immunological methods [15]. NATOG is a neurotransmitter-derived metabolite, which was secreted into the human system by the parasite, *Onchocerca volvulus*. This compound was identified as a biomarker for onchocerciasis by metabolomics, an approach to identify and quantify overall metabolites in biological samples [16]. With the high sensitivity and discovery power of metabolomics, it is an excellent tool for pinpointing novel biomarkers of infectious diseases, including schistosomiasis [17]. Balog, *et al*. investigated urine markers for *S*. *mansoni* infection in people who lived in endemic areas of Uganda and found some metabolites that showed biomarker potential, i.e., acetate, citrate, dimethylamine, guanidino acetate [18]. In addition, Adebayo, *et al*. studied levels of metabolite changes in blood and urine of volunteers who lived in southwestern Nigeria, where *S*. *haematobium* is common. They found that levels of many metabolites, such as, phosphatidylcholine in blood and catechol in urine could be used as markers of urogenital schistosomiasis [19]. Hu, *et al*. performed metabolomic analysis of blood and urine samples of *S*. *japonicum-*infected mice. They found that metabolite profiles of the mice were altered earlier than parasite egg production, and several blood biomarkers, including phosphatidylcholine and colfosceril, as well as urine biomarkers like xanthurenic acid and naphthalenesulfonic acid, were proposed [20]. Furthermore, Chienwichai, *et al*. examined serum metabolomics from *S*. *mekongi*-infected mice and identified markers of early Mekong schistosomiasis, for example, heptadecanoyl ethanolamide, picrotin, and theophylline [21].

Although metabolomics is an exceptional tool for biomarker discovery, there are a number of issues that previous research did not explore. First of all, all studies identified biomarkers based on infection of only one species [18–21]. A lack of variety in parasite species may hinder the generalization of the discovered markers, especially for intestinal schistosomiasis that caused by 5 species of *Schistosoma* worms. Furthermore, only some studies focused on identifying markers of early schistosomiasis. Pathobiology of *Schistosoma* infection is related with egg production of the flukes [8]. The classic parasitological examination relies on detection of eggs excreted from host body, indicating that damages have already occurred to the host. Using markers to detect the infection earlier than the production of parasite eggs would prevent harmful events to people and reduce disease spreading. Up until currently, there are only 4 studies those attempted to discover metabolite biomarkers of early schistosomiasis, which performed in *S*. *mansoni*, *S*. *japonicum*, and *S*. *mekongi-*infected subjects [20–23]. Unfortunately, none of the 4 studies proposed overlapping markers, which supports our earlier statement that elucidating markers for *Schistosoma* infection in different species may be challenging for the further development of schistosomiasis markers. In addition to the discovery of markers, data from metabolomics can be used to explore disease progression at the different points of time [21,23]. Understanding the progression will provide insight into pathogenesis as well as biology of the parasite. It is clear that eggs of *Schistosoma* parasites induce granulomatous inflammation to the intestine [8]. In the contrary, the information regarding intestinal environment of early schistosomiasis has never been investigated before. To cope with the aforementioned issues, we aimed to identify early biomarkers of intestinal *Schistosoma* infection caused by 3 different species and progression of intestinal pathology since the early stage. Here in this study, we infected mice with *S*. *mansoni*, *S*. *japonicum*, and *S*. *mekongi*, the 3 species those have different geographical distribution [24]. Africa, Central and South America are predominantly affected by *S*. *mansoni*, while East Asia and The Philippines have *S*. *japonicum* as the major species. Finally, Laos and Cambodia are the only areas where *S*. *mekongi* is restricted [24]. By choosing these 3 species, we have covered the worldwide distribution of *Schistosoma* flukes. Additionally, we collected their fecal samples at pre-, 1-, 2-, 4-, and 8-weeks post-infection (PI), in order to identify biomarkers of early intestinal schistosomiasis and investigate disturbances of gut metabolic pathway though the course of infection. Types and intensities of metabolites in fecal samples were studied using an untargeted metabolomic approach. Findings of our study would be beneficial for future development of schistosomiasis markers those can be used regardless of infected species and geographical distribution, leading to lower losses from this parasite.

## Results

### Fecal metabolite levels changed since the first week of Schistosoma infection

**Fig 1:**
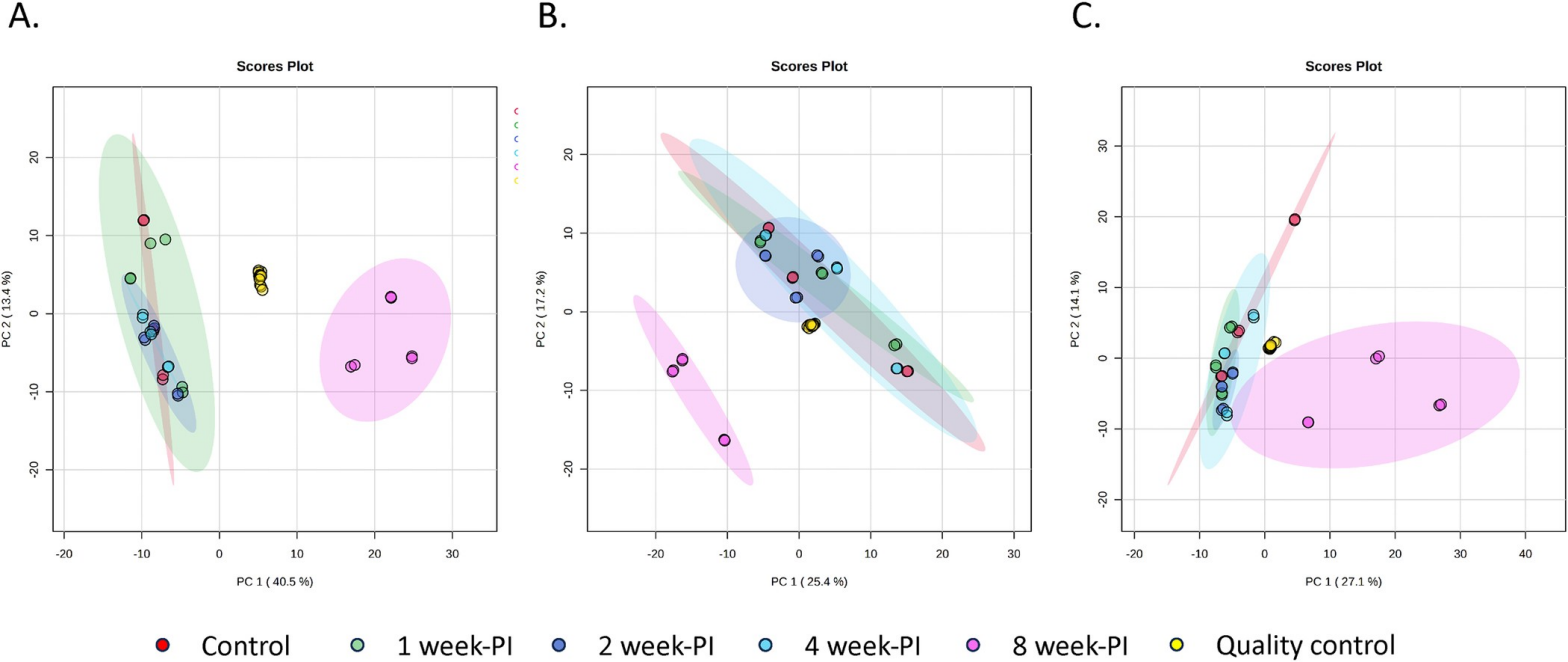
Principal component analysis (PCA) plots for quality control of metabolomic data. From the plots, QC samples (yellow dots) are clustered tightly in the center of the plots, indicating reliability of MS systems. Red, green, navy, blue, and pink dots refer to control, 1-week, 2-week, 4-week, and 8-week PI groups, respectively. A). *S*. *mansoni* infection. B). *S*. *japonicum* infection C). *S*. *mekongi* infection.

Three groups of ICR mice were infected with *S*. *mansoni*, *S*. *japonicum*, and *S*. *mekongi*. The mice were observed for signs and symptoms of the disease for 8 weeks. In addition, feces of mice were collected at pre-, 1-, 2-, 4-, and 8 weeks post infection for untargeted metabolomic study. No mice showed changes in body weight, food consumption, behavior, or symptoms of intestinal schistosomiasis. With modified Kato-Katz method, eggs of all three *Schistosoma* spp. were not observed in the feces of mice earlier than 8 weeks PI. Biomarkers identified at 1, 2 and 4 weeks PI were considered as markers of early disease. All data were assessed for their quality before further analysis (S1–S3 Files). All metabolomic data met the predefined criteria stating that all quality control samples must be clustered tightly in the center of principal component analysis plots (Fig 1). This finding reflected the reliability and reproducibility of the data, and further analysis proceeded.

**Fig 2:**
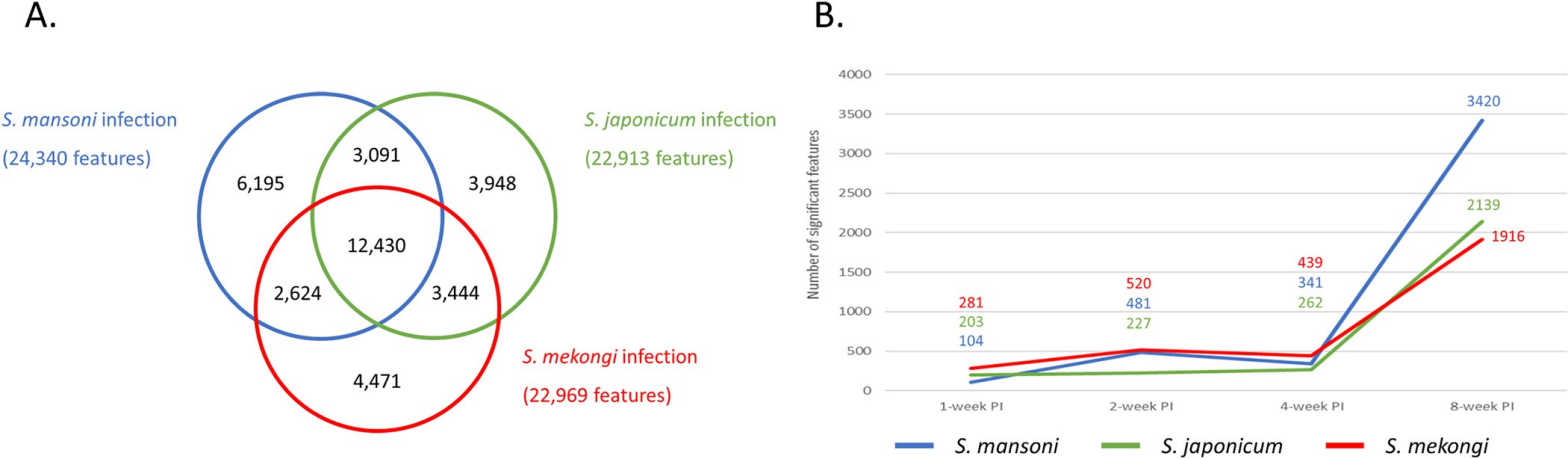
Profiles of metabolomic data from feces of mice infected with *Schistosoma mansoni*, *Schistosoma japonicum* or *Schistosoma mekongi*. A. Venn diagram depicts numbers of features identified from each species. B. Line chart shows numbers of significantly changed features from each species at the different time-points. The blue circle/line represents data from *S*. *mansoni-*infected mice. The green circle/line represents data from *S*. *japonicum-*infected mice. The red circle/line represents data from *S*. *mekongi-*infected mice.

Fecal metabolomics identified 24,340, 22,913 and 22,969 features in the mice infected with *S*. *mansoni*, *S*. *japonicum* or *S*. *mekongi*, respectively (Fig 2A). After removal of duplicates, there were 36,203 unique features; 12,430 (34.33%) of which were shared among mice infected with different *Schistosoma* spp. These shared features were our targets for in-depth analysis of trans-genus biomarkers. To investigate features that had changed significantly at the different time points in each infection group, fold change and *p* value of all features were calculated by comparison with the control group, the sex-matched mice from which the feces were collected before infection occurred. The abundance of hundreds of features was significantly altered as early as 1 week after infection by all *Schistosoma* spp. (Fig 2B). The number of significantly changed features increased over time and sharply rose at 8 weeks PI, coinciding with the presence of parasite eggs. These findings correlated well with the pathogenesis of intestinal schistosomiasis, which involved induction of intestinal injury by parasite eggs.

**Fig 3:**
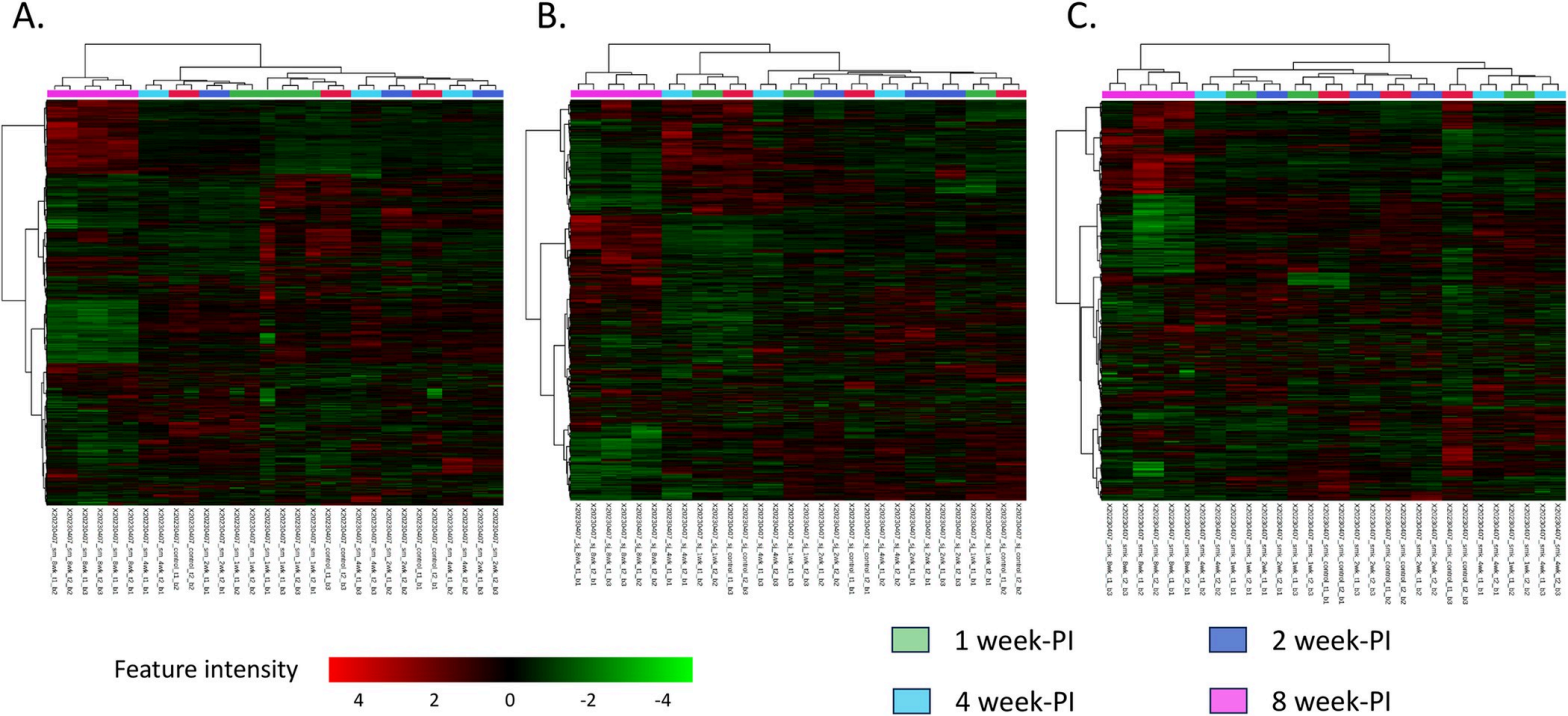
Hierarchical clustering heatmap analysis of metabolomic data from control, and 1-, 2-, 4- and 8 week-PI groups infected with *Schistosoma* spp. A. Data from *S*. *mansoni-*infected mice. B. Data from *S*. *japonicum-*infected mice. C. Data from *S*. *mekongi-*infected mice. Green bar represents data from 1 week-PI group. Navy bar represents data from 2 week-PI group. Blue bar represents data from 4 week-PI group. Pink bar represents data from 8 week-PI group. Red color indicates features with increased intensity and green color indicates features with decreased intensity.

**Fig 4:**
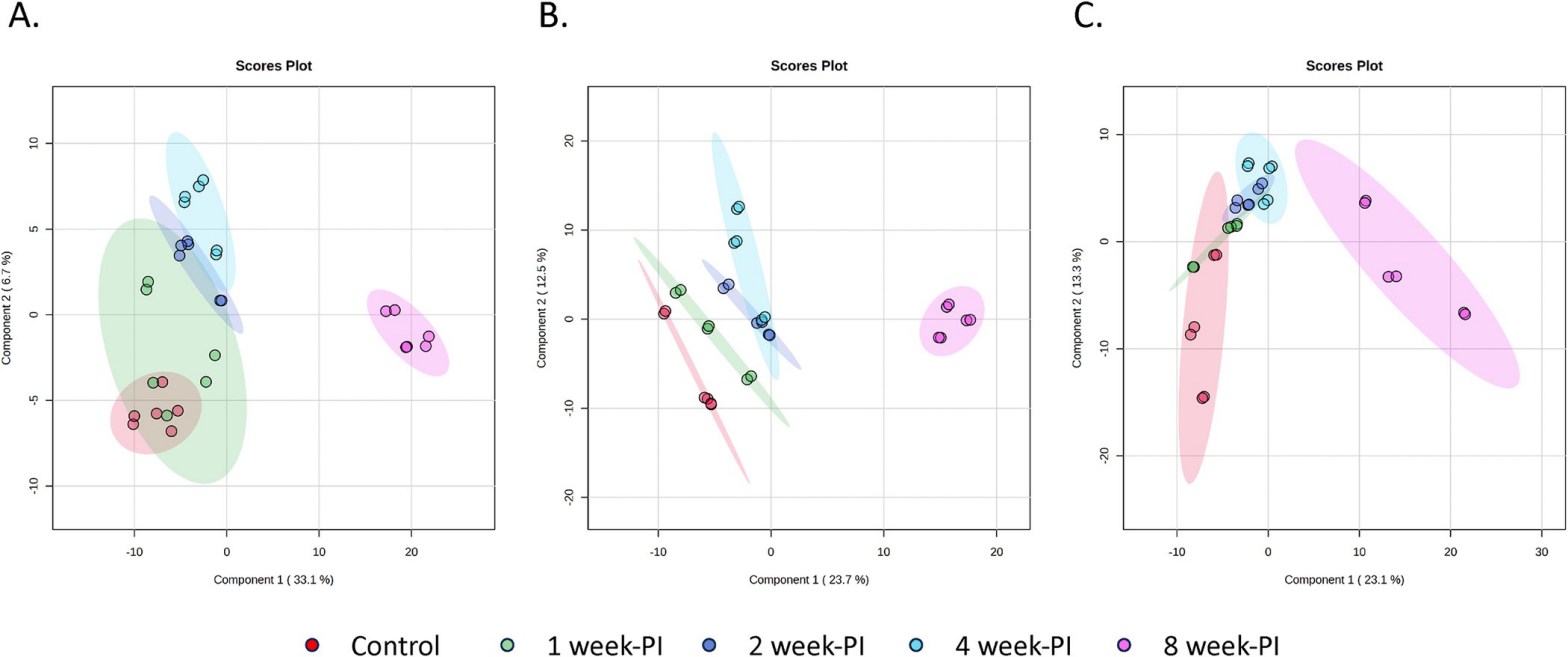
Partial least squares-discriminant analysis of metabolomic data from control, and 1-, 2-, 4- and 8 week-PI groups infected with *Schistosoma* spp. A. Data from *S*. *mansoni-*infected mice. B. Data from *S*. *japonicum-*infected mice. C. Data from *S*. *mekongi-*infected mice. Red dots represent data from the control group. Green dots represent data from the 1 week-PI group. Navy dots represent data from the 2 week-PI group. Blue dots represent data from the 4 week-PI group. Pink dots represent data from the 8 week-PI group. Ellipses represent 95% confidence region.

To investigate further the pattern of significantly altered features together with disease progression, hierarchical clustering heatmaps were generated from metabolomic data. With the clustering applied, data from the 8 week-PI group (pink bar) were clearly clustered, separated from data of other duration in all species (Fig 3). The color scheme of red (increased level) and green (decreased level), this finding showed the distinct perturbation of metabolite intensities at this infection time point. The patterns of altered features on heatmaps were similar for data from the different species, indicating the same disease progression for infection with different *Schistosoma* spp. In addition, supervised multivariate analysis, partial least squares-discriminant analysis (PLS-DA), was used to explore separation of data among infection time points. The PLS-DA model yielded results consistent with those observed in the heatmaps. For all species, the dataset of the 8 week-PI group (pink dots) was distinctly separated from the other datasets. The datasets of the control (red dots), 1 week-PI (green dots), 2 week-PI (navy dots), and 4 week-PI (blue dots) groups were close, but a noticeable separation was still evident. Over time, the dots from the infected groups gradually separated from those of the control group (Fig 4A–4C). The variable importance in projection (VIP) score of the PLS-DA model was calculated to select the features that could be applied for discrimination between the control and infection groups. The top 10 features with highest VIP score in all *Schistosoma* infections are presented in Table 1.

**Table 1:** Top 10 putative fecal metabolites with highest VIP score from PLS-DA model.

No.	Median M/Z	Median retention time (minute)	Ionization mode	Putative metabolite	METLIN ID	Chemical Formular	Ion adducts	Mass error (ppm)	VIP score		
									*S*. *mansoni*	*S*. *japonicum*	*S*. *mekongi*
1.	520.3993	10.34	Positive	C-12 NBD Ceramide	45025	C_36_H_61_N_5_O_6_	M+Na-(C_6_H_12_O_6_-H_2_O)	0	2.1026	1.9761	1.7822
2.	467.3930	12.65	Positive	Staphidine	67146	C_42_H_58_N_2_O	M+Na-(C_6_H_12_O_6_-H_2_O)	0	1.9839	1.912	1.794
3.	918.6907	9.07	Positive	Phosphatidylethanolamine (PE) (21:0/24:0)[U]	40478	C_50_H_100_NO_8_P	M+2Na-H	1	1.8447	2.0463	1.7915
4.	449.3628	12.64	Positive	Chlorogenin	84237	C_27_H_44_O_4_	M+H	0	1.8081	1.935	1.7259
5.	459.3472	10.09	Positive	18-acetoxy-1α-hydroxy vitamin D3	42339	C_29_H_46_O_4_	M+H	1	1.7253	1.9873	1.806
6.	445.3319	13.74	Negative	Trihydroxycoprostanoic acid	57683	C_28_H_48_O_5_	M-H_2_O-H	0	1.6716	2.0368	1.8926
7.	255.1956	12.03	Positive	3-Nor-3-Oxopanasinsan-6-ol	44489	C_14_H_22_O_2_	M+CH_3_OH+H	0	2.0529	1.8732	1.6763
8.	377.2694	8.75	Negative	13,14-dihydro-15-keto PGF2α isopropyl ester	45685	C_23_H_40_O_5_	M-H_2_O-H	0	1.879	1.6935	2.1015
9.	375.2537	10.98	Negative	Prostaglandin E2 isopropyl ester	45413	C_23_H_38_O_5_	M-H_2_O-H	0	1.6371	1.6034	1.7281
10.	919.7000	9.09	Positive	Phosphatidylglycerol (PG) (22:0/22:2(13Z,16Z))	79548	C_50_H_95_O_10_P	M+CH_3_OH+H	0	1.5994	2.3064	1.8331

**Fig 5:**
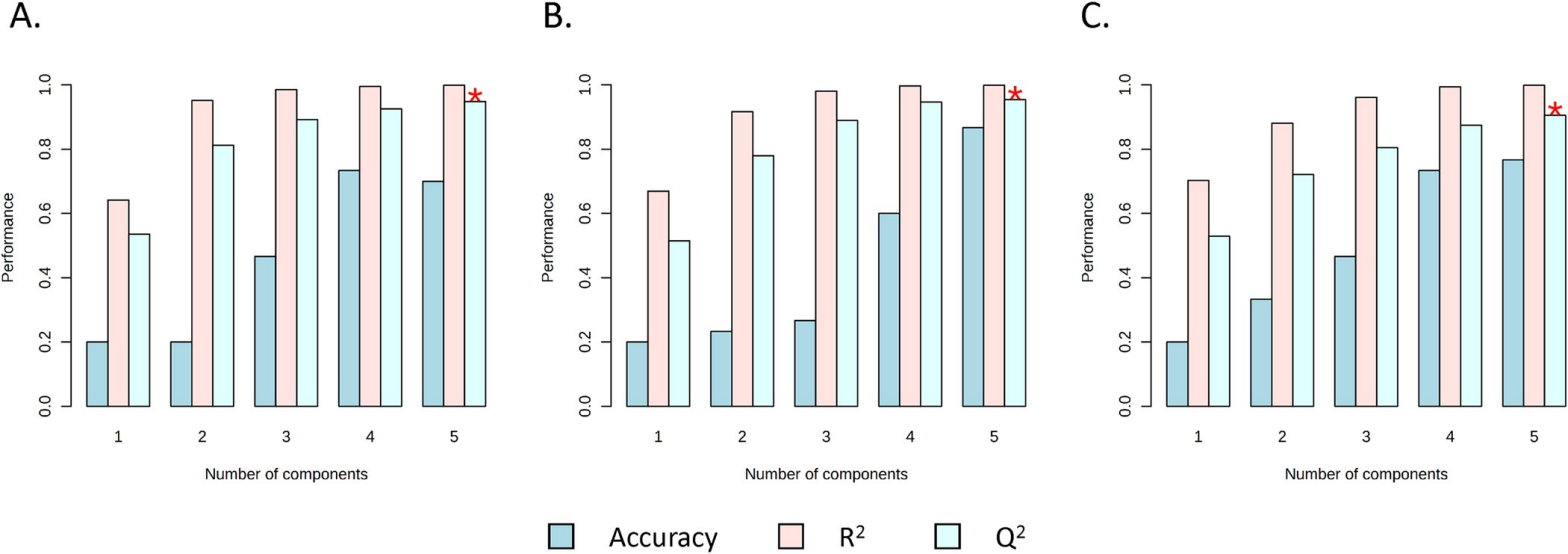
The PLS-DA model performance evaluation using leave-one-out cross-validation (LOOCV) method. Q^2^ values of all models were over 0.8, indicated that the model was the good predictor and less likely for overfitting A). *S*. *mansoni* infection. B). *S*. *japonicum* infection C). *S*. *mekongi* infection.

Performance of the PLS-DA model was evaluated using the leave-one-out cross-validation method. The Q^2^ scores of the model in all infection groups were >0.8, indicating good prediction of the model and less likelihood of overfitting (Fig 5). To confirm the separation at the early stage, the PLS-DA model was used to explore the separation in pairwise manner for the control group and each time point of infection for all *Schistosoma* spp. Even at 1 week PI, the separation was evident (S1A Fig), indicating that the early stage in *Schistosoma* infection can result in changes to fecal metabolites. Subsequently, pathway analysis was conducted to explore the cellular pathways affected by this parasitic infection.

### Changes in fecal metabolites reflected alteration of lipid metabolism, glycan biosynthesis and metabolism pathways

**Fig 6:**
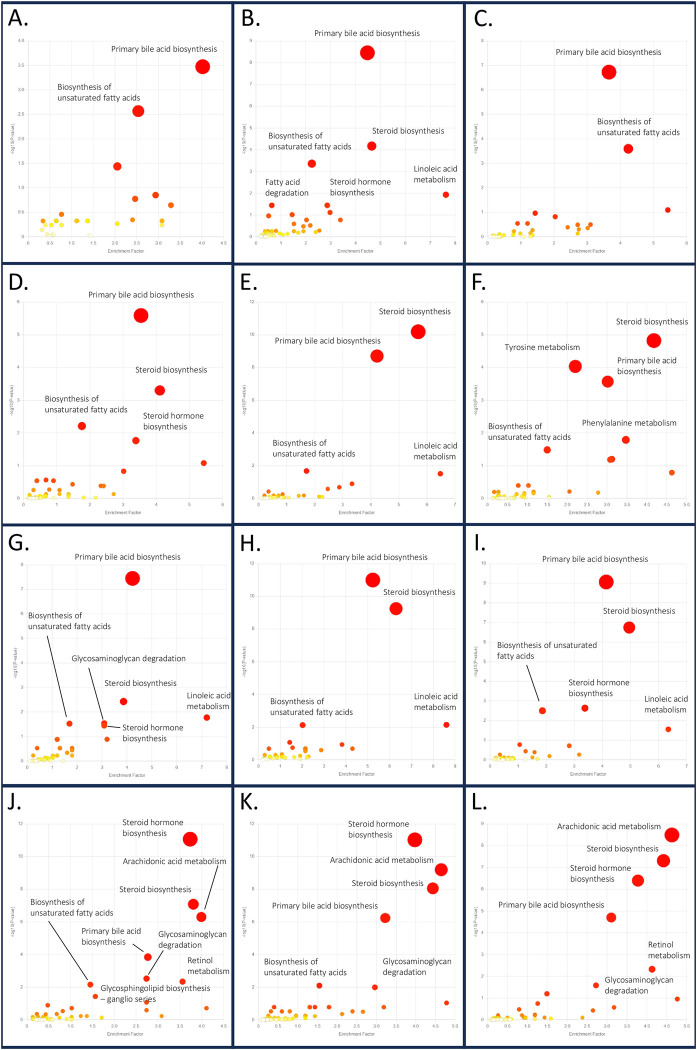
Pathway analysis of metabolomic data from 1-, 2-, 4- and 8 week-PI groups infected with *Schistosoma* spp. A. Data from *S*. *mansoni-*infected mice at 1 week PI. B. Data from *S*. *japonicum-*infected mice at 1 week PI. C. Data from *S*. *mekongi-*infected mice at 1 week PI. D. Data from *S*. *mansoni-*infected mice at 2 weeks PI. E. Data from *S*. *japonicum-*infected mice at 2 weeks PI. F. Data from *S*. *mekongi-*infected mice at 2 weeks PI. G. Data from *S*. *mansoni-*infected mice at 4 weeks PI. H. Data from *S*. *japonicum-*infected mice at 4 weeks PI. I. Data from *S*. *mekongi-*infected mice at 4 weeks PI. J. Data from *S*. *mansoni-*infected mice at 8 weeks PI. K. Data from *S*. *japonicum-*infected mice at 8 weeks PI. L. Data from *S*. *mekongi-*infected mice at 8 weeks PI.

To highlight the affected pathways caused by *Schistosoma* infection, the Mummichog algorithm and KEGG pathway database of mice (*Mus musculus)* were applied through Metaboanalyst platform. The pathway analysis was performed by comparing metabolomic profiles of the control and *Schistosoma*-infected groups at the different time points after infection. The three *Schistosoma* spp. cause similar clinical manifestations in patients; hence, we focused on the affected pathways that were highlighted in all species. At 1 week PI, primary bile acid biosynthesis was the only common significantly affected pathway in all *Schistosoma* spp. At 2 weeks PI, the primary bile acid biosynthesis, steroid biosynthesis, and biosynthesis of unsaturated fatty acids pathways were significantly affected in all *Schistosoma* spp. At 4 weeks PI, there were four enriched pathways: primary bile acid biosynthesis, steroid biosynthesis, biosynthesis of unsaturated fatty acids, and linoleic acid metabolism. Lastly, primary bile acid biosynthesis, steroid biosynthesis, steroid hormone biosynthesis, arachidonic acid metabolism, and glycosaminoglycan (GAG) degradation pathways were significantly affected at 8 weeks PI for all three *Schistosoma* spp. (Fig 6 and S1 Table). The scatter plot patterns from *S*. *japonicum* (Fig 6B, 6E, 6H, and 6K) and *S*. *mekongi* (Fig 6C, 6F, 6I and 6L) looked more similar to each other than to the pattern from *S*. *mansoni* (Fig 6A, 6D, 6G, and 6J). These findings provide some clues regarding the intestinal pathogenesis of the disease.

**Fig 7:**
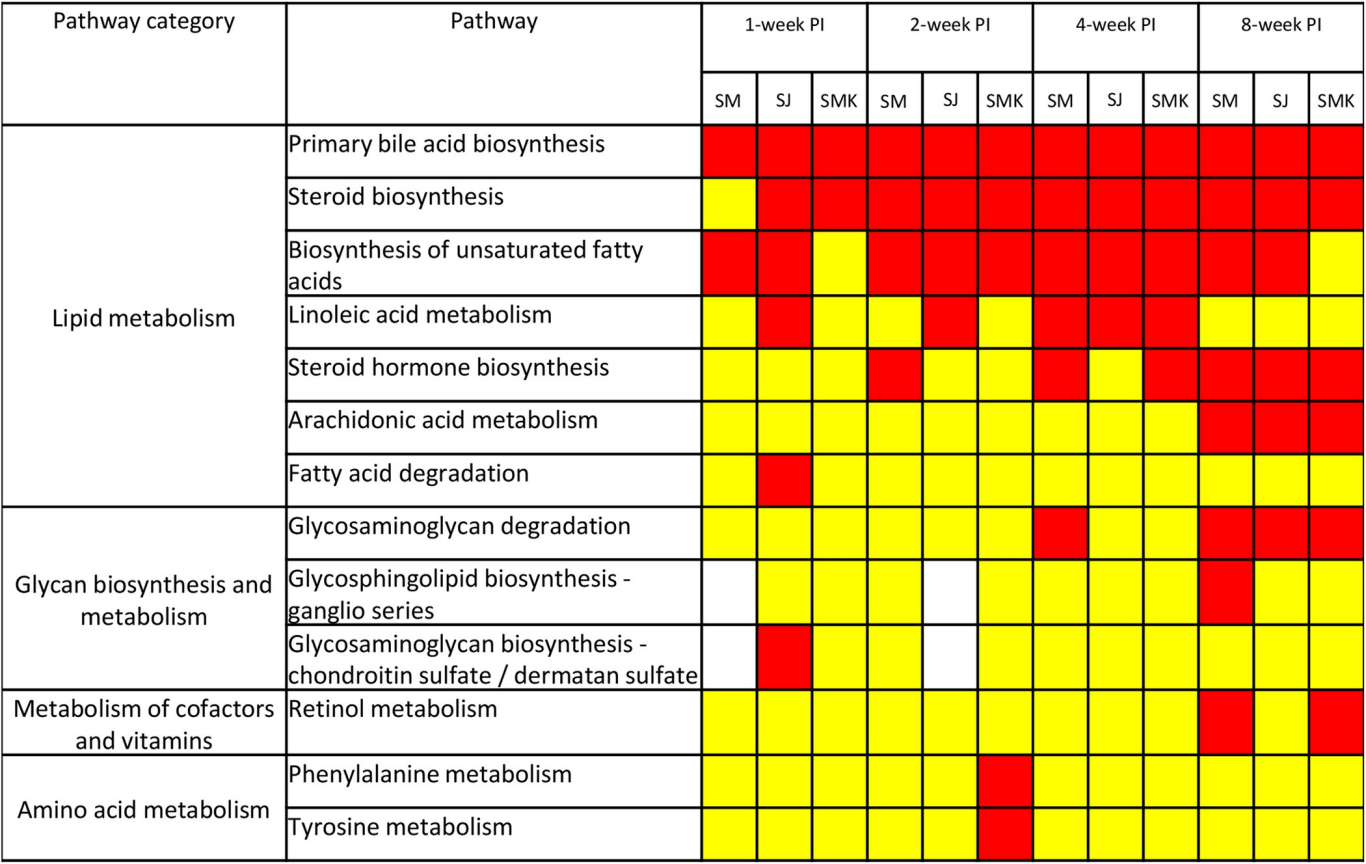
Arrangement of the affected pathways according to KEGG database. The affected pathways were categorized by pathway class. Yellow represents enriched pathways, while red represents enriched pathway with *p*<0.05.

When all enriched pathways were categorized according to the KEGG database, it was clear that all significantly affected pathways in early infection were classified into the lipid metabolism category (Fig 7). This indicates that *Schistosoma* infection, even in the absence of parasite eggs, can affect the host’s intestinal environment and potentially influence the disease pathogenesis. The glycosaminoglycan degradation pathway was significantly enriched at 8 weeks PI. In addition to this pathway, several glycan biosynthesis and metabolism pathways showed enrichment, although not significantly. The role of glycans in the pathogenesis of intestinal schistosomiasis has been rarely explored, making it an interesting topic for further discussion.

### Vitamin D analogs proposed as potential trans-genus fecal biomarkers of early schistosomiasis

**Fig 8:**
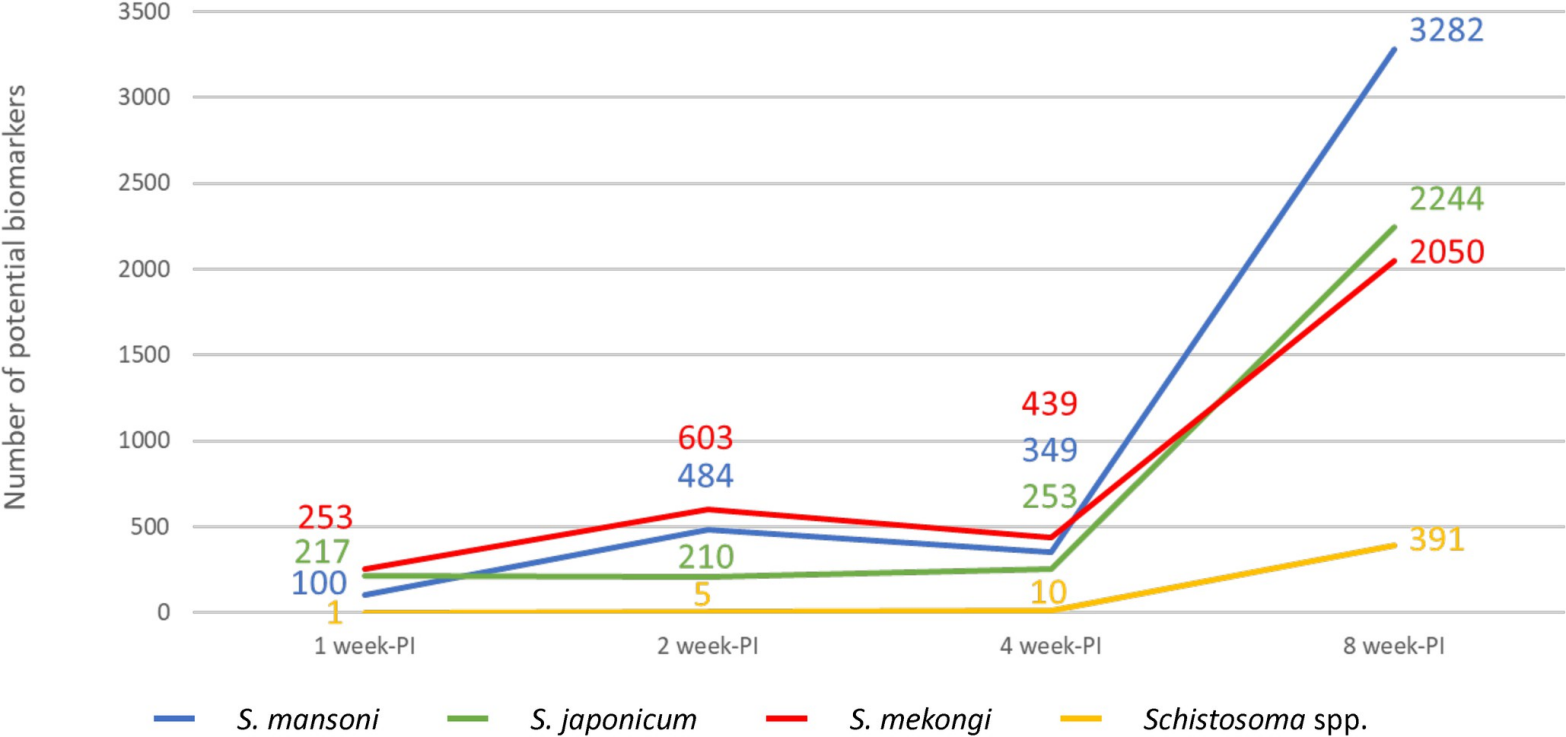
Numbers of features that met the criteria for receiver operating characteristic curve analysis. The blue line represents data from *Schistosoma mansoni-*infected mice. The green line represents data from *Schistosoma japonicum-*infected mice. The red line represents data from *Schistosoma mekongi-*infected mice. The yellow line represents data from *Schistosoma* spp.-infected mice.

Trans-genus biomarkers were identified using the classical univariate receiver operating characteristic (ROC) curve analysis in combination with VIP score from the PLS-DA model. All features from the four time points of infection for each *Schistosoma* spp. were compared with their control counterparts in a pairwise manner. The criteria for further analysis of potential biomarkers were area under the ROC curve >0.8, fold change ≥2, and *p*<0.05. The patterns of significantly changed features (Fig 2B) and features that met these criteria (Fig 8) were similar, increasing over time. When we focused on the features shared among all *Schistosoma* spp. (yellow line), there were 1, 5, 10 and 391 at 1, 2, 4 and 8 weeks PI, respectively. The putative metabolites of the features are shown in Table 2.

**Table 2:** Putative metabolites that met the criteria for receiver operating characteristic curve analysis.

No.	Median M/Z	Median retention time (minute)	Ionization mode	Putative metabolite	METLIN ID	Chemical Formular	Ion adducts	Mass error (ppm)	*S*. *mansoni*			*S*. *japonicum*			*S*. *mekongi*		
									ROC-AUC	*p*-value	FC	ROC-AUC	*p*-value	FC	ROC-AUC	*p*-value	FC
1 week-PI																	
1.	631.6054	17.87	Positive	Sphingomyelin (SM) (d18:2/24:1)	83780	C_47_H_91_N_2_O_6_P	M+H-C_6_H_12_O_6_	1	0.80	0.029	-2.54	0.94	0.003	-2.86	0.83	0.024	-2.97
2 week-PI																	
1.	413.3410	12.65	Positive	25-Hydroxyvitamin D2	217	C_28_H_44_O_2_	M+H	1	1	0.001	2.29	1	0.003	-4.78	1	0.021	-3.19
2.	771.5706	8.76	Positive	Phosphatidic acid (PA) (22:1(11Z)/22:4(7Z,10Z,13Z,16Z))	81919	C_47_H_83_O_8_P	M+H-2H_2_O	1	1	0.003	-3.59	0.89	0.014	-3.44	1	0.040	-4.10
3.	493.2813	6.18	Positive	Phe Phe Val Val	137437	C_28_H_28_N_2_O	M+H-H_2_O	0	1	0.000	-6.06	1	0.001	-3.25	1	0.044	-3.08
4.	585.3795	12.23	Negative	Ganoderic acid Md	90631	C_35_H_54_O_7_	M-H	0	1	0.000	-2.29	0.89	0.004	-2.11	1	8.30E^-06^	-2.90
5.	509.2880	13.10	Negative	PG (18:1(9Z)/0:0)	40878	C_24_H_47_O_9_P	M-H	1	1	0.022	-8.81	0.83	0.021	-2.63	1	0.025	-13.48
4 week-PI																	
1.	455.3483	13.10	Negative	Unidentified feature	-	-	-	-	0.88	0.042	-2.13	1	0.014	-3.76	1	1.75E^-05^	-35.17
2.	456.3516	13.37	Negative	Unidentified feature	-	-	-	-	0.97	0.003	-5.97	1	0.003	-9.05	1	0.028	-5.45
3.	739.5989	12.69	Negative	Unidentified feature	-	-	-	-	0.88	0.016	-3.32	1	0.001	-8.80	1	1.04E^-06^	-33.11
4.	455.3511	12.81	Negative	1α-hydroxy-2β-(3-hydroxypropoxy) vitamin D3	42398	C_27_H_44_O_4_	M-H_2_O-H	3	0.94	0.008	-4.54	1	0.001	-8.74	1	0.016	-5.36
5.	426.3665	19.76	Negative	Unidentified feature	-	-	-	-	0.83	0.012	-2.47	1	0.010	-3.19	1	0.000	-8.95
6.	453.3941	17.38	Negative	Unidentified feature	-	-	-	-	0.86	0.036	-2.03	1	0.002	-2.67	1	0.004	-4.23
7.	525.2883	19.76	Negative	Asp Arg Arg Val	125377	C_21_H_40_N_10_O_7_	M-H_2_O-H	3	0.83	0.019	-2.04	1	0.027	-2.70	1	0.000	-6.35
8.	451.3785	19.71	Negative	Unidentified feature	-	-	-	-	0.88	0.008	-2.81	0.88	0.020	-3.02	1	0.000	-9.39
9.	452.3819	19.71	Negative	Unidentified feature	-	-	-	-	0.90	0.004	-2.82	0.94	0.026	-3.13	1	0.000	-9.35
10.	437.3629	18.64	Negative	Unidentified feature	-	-	-	-	1	0.004	-3.97	0.86	0.013	-2.91	1	0.000	-11.09
8 week-PI (Only top-10 features were shown).																	
1.	255.1956	12.03	Positive	3-Nor-3-Oxopanasinsan-6-ol	44489	C_14_H_22_O_2_	M+CH_3_OH+H	0	1	4.65E^-12^	47.23	1	1.44E^-05^	8.12	1	0.006	6.43
2.	972.6220	9.47	Negative	Unidentified feature	-	-	-	-	1	4.48E^-11^	-30.72	1	1.66E^-08^	-11.50	1	6.68E^-05^	-5.69
3.	251.2368	11.13	Positive	(+)-12-(2-Cyclopenten-1-yl)-2-dodecanone	70928	C_17_H_30_O	M+H	0	1	5.99E^-11^	69.84	1	5.96E^-05^	4.37	1	2.79E^-05^	7.58
4.	502.3249	9.67	Negative	Unidentified feature	-	-	-	-	1	1.06E^-10^	-6.64	1	2.87E^-07^	-4.26	1	0.000	-2.95
5.	459.3472	9.09	Positive	1α,25-dihydroxy-2β-(2-hydroxyethoxy)vitamin D3	42369	C_29_H_48_O_4_	M+H-H_2_O	0	1	1.62E^-10^	-8.21	1	6.63E^-06^	-3.08	1	0.001	-2.14
6.	918.6907	9.07	Positive	PE(21:0/24:0)[U]	40478	C_42_H_82_NO_8_P	M+2Na-H	1	1	1.68E^-10^	-65.83	1	5.15E^-05^	-5.58	1	0.000	-7.76
7.	582.3424	9.48	Positive	Ciclesonide	85511	C_32_H_44_O_7_	M+ACN+H	0	1	1.77E^-10^	-6.68	1	2.02E^-10^	-4.72	1	2.79E^-05^	-2.94
8.	452.3370	11.00	Positive	(24R)-1α,24,25-trihydroxy-22-oxavitamin D3	42005	C_27_H_44_O_4_	M+NH_4_	0	1	1.91E^-10^	-3.94	1	3.36E^-06^	-5.17	1	0.001	-2.64
9.	375.2511	12.42	Positive	Asp Phe Lys Lys	121248	C_25_H_40_N_6_O_7_	M+H-(C_6_H_12_O_6_-H_2_O)	0	1	3.04E^-10^	10.59	1	0.003	2.83	1	0.006	3.12
10.	847.6110	9.08	Negative	Unidentified feature	-	-	-	-	1	3.80E^-10^	-13.51	1	4.2E^-07^	-6.06	1	6.02E^-05^	-8.58

**Fig 9:**
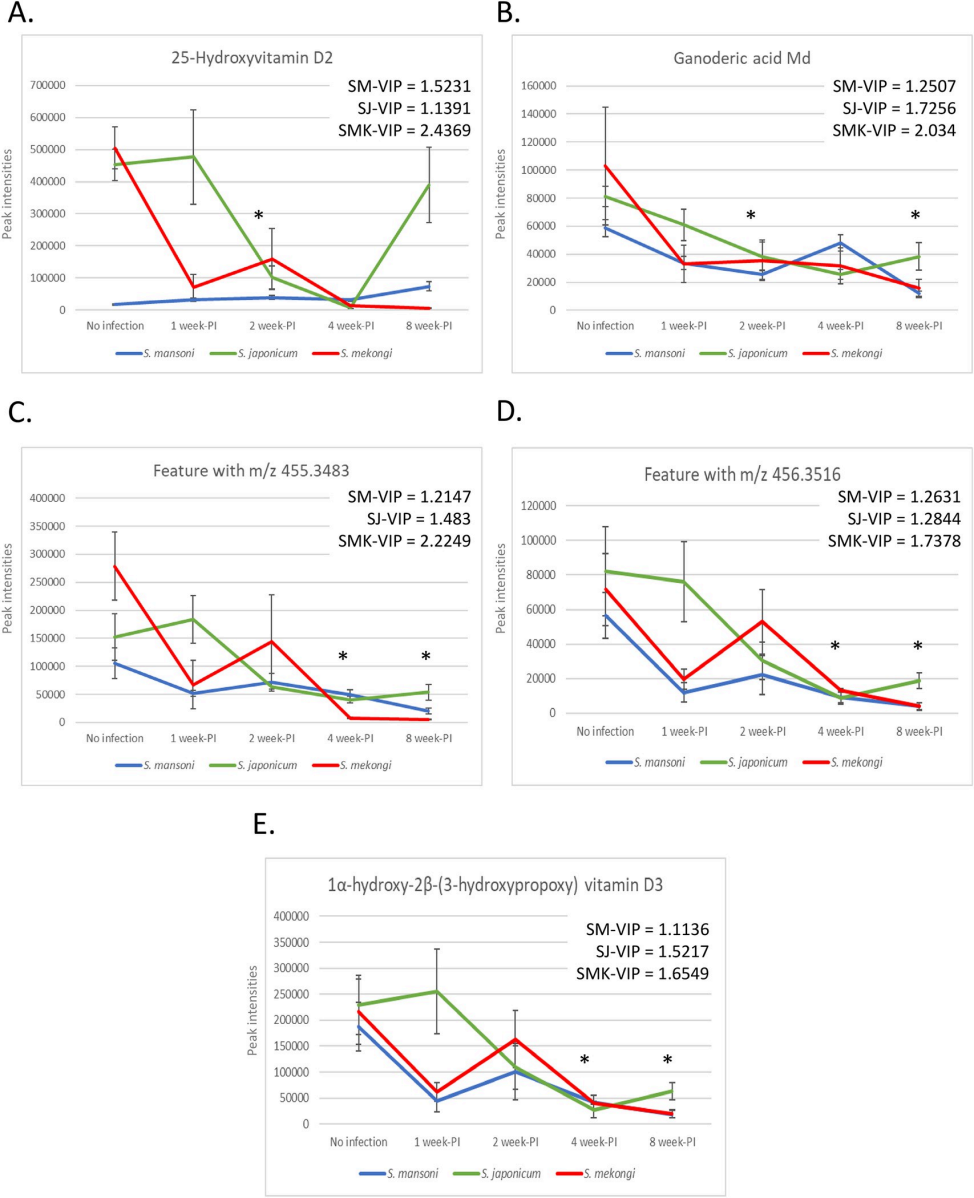
Potential trans-genus fecal biomarkers for early intestinal schistosomiasis. A. 25-Hydroxyvitamin D2 B. Ganoderic acid Md C. Feature with m/z 455.3483 D. Feature with m/z 456.3516 E. 1α-hydroxy-2β-(3-hydroxypropoxy) vitamin D3. Asterisk means statistical difference.

The potential biomarkers of early schistosomiasis, described as the features that met the area under the ROC curve criteria and had a VIP score ≥1 for early infection with all *Schistosoma* spp., were highlighted. No potential biomarkers were present at 1 week PI, while the 2 week-PI group showed two potential biomarkers, 25-hydroxyvitamin D2 and ganoderic acid Md (Fig 9A and 9B). At 4 week-PI, three putative metabolites, the unidentified feature with m/z 455.3483, the unidentified feature with m/z 456.3516, and 1α-hydroxy-2β-(3-hydroxypropoxy) vitamin D3, were highlighted (Fig 9C–9E). Two vitamin D derivatives, 25-hydroxyvitamin D2 and 1α-hydroxy-2β-(3-hydroxypropoxy) vitamin D3, were potential biomarkers. These vitamin D analogs are part of the lipid metabolism pathway, and their presence as potential biomarkers might be related to the affected pathways identified earlier. Therefore, we suggest five molecules, including vitamin D derivatives, as potential trans-genus markers of early intestinal schistosomiasis.

## Discussion

Intestinal schistosomiasis affects vast numbers of the global population. Much research has gone into reducing losses from this parasitic infection; however, the numbers of cases and deaths remain high. Identification of markers for early detection of the disease could help with reduction of clinical symptoms and prevent spread of the disease. Unfortunately, previous studies investigated only infection with one species of *Schistosoma*, leaving a substantial gap for generalization of identified markers. Additionally, no study focused on progression of intestinal disease. In the present study, we collected fecal samples from mice infected with *S*. *mansoni*, *S*. *japonicum*, or *S*. *mekongi*. The feces were collected before the parasitological analysis could detect the infection, which is the standard protocol for diagnosing intestinal schistosomiasis. Biomarkers in feces were identified using metabolomics and appropriate bioinformatic tools. We identified biological pathways related to disease progression and five compounds with the potential for development as trans-genus biomarkers for early intestinal schistosomiasis.

We performed our experiments by collecting feces of mice before, and 4 time points after 3 species of blood fluke infection. We followed the same mice and collected their feces throughout the course of the infection to reduce variation caused by genetic diversity of mice. Literatures suggest that the duration of 2 months, the relatively short period in their lifespan, was less likely to affect their normal physiology and metabolite moiety in their body. The substantial shift of metabolites within the mouse body were reported when their age increased by 10–12 months [25–26]. These findings support the integrity of our study design for the metabolomic comparison of control and infected groups.

Our findings correlated well with the pathogenesis of intestinal schistosomiasis. The main pathogenic agent of schistosomiasis is parasite eggs, which usually present at 5 weeks after infection. Before 5 weeks, patients may develop acute schistosomiasis, which is characterized as an immunological response to penetrating cercaria [8]. Our findings demonstrated that the number of significantly changed features from feces during the early period were low (104–520), when compared with the period when eggs were present (1,916–3,420). This indicates that the pathobiology of acute schistosomiasis affects the intestinal environment to a lesser degree compared with chronic schistosomiasis. Li *et al*. was the only study to conduct fecal metabolomics in schistosomiasis. They collected fecal samples of *S*. *mansoni-*infected mice as early as 13 days after infection, however, they did not found the alteration during the early stage [22]. They found changes in several amino acids, short-chain fatty acids (SCFA), and other metabolites at 53 days PI. In comparison with our study, we found many altered putative metabolites since the first week after infection. Although some putative metabolites were in the same group, i.e. fatty acid molecules, we identified a wider range of putative metabolites than study of Li *et al*. Regarding metabolomics from serum samples, most previous studies found low numbers of altered metabolites at the early stage. Huang *et al*. identified 47–58 important metabolites for detecting infection during the first 5 weeks following exposure to *S*. *japonicum* cercariae in mice. Unfortunately, their study did not extend to the late stages of schistosomiasis, making it impossible to compare with the number of metabolites during the presence of parasite eggs [23]. Our previous study on *S*. *mekongi* infection in mice found 76–91 significantly changed metabolites during 2–4 weeks PI. After parasite eggs were present at 8 weeks PI, the number of altered metabolites increased to 359 [21]. These findings suggest that parasite egg production is the major factor influencing the number of altered metabolites in the host. However, the levels of some metabolites were changed before the presence of parasite eggs, indicating their potential as biomarkers of early schistosomiasis.

We found that many lipid molecules were highlighted as important features for discrimination of *Schistosoma* infection by PLS-DA, for example, C-12 NBD Ceramide, Phosphatidylethanolamine (PE) (21:0/24:0)[U], Phosphatidylglycerol (PG) (22:0/22:2(13Z,16Z)) (Table 1). The fecal metabolite profiling studies of helminthic infection also reported changes in many fatty acids and lipid -related molecules. Jenkins, *et al*. conducted metabolomic study in feces of *Strongyloides stercoralis-*infected people and found that SCFA, for example, propionate, butyrate, were one of the prominent molecules those changed their levels in infected group [27]. Moreover, Saric, *et al*. performed nuclear magnetic resonance (NMR)-based metabolomics from plasma, urine, and feces of mice infected with *Echinostoma caproni*, the intestinal flukes. In fecal metabolomics, levels of some SCFA, i.e., acetate, butyrate, were changed in infected mice [28]. In schistosomiasis, Li, *et al*. also reported changes in SCFA following *S*. *mansoni* infection [22], indicating that fatty acids and lipid molecules may play a key role in intestinal environment of the parasite. Our pathway analysis results supported the earlier findings that many pathways in lipid metabolism category were identified as altered pathways. Some pathways, for example, primary bile acid biosynthesis, steroid biosynthesis, biosynthesis of unsaturated fatty acids, were highlighted as early as 1 week-PI and remained altered throughout the course of infection (Fig 7). Therefore, we investigated further to postulate the ongoing process of intestinal schistosomiasis using pathway analysis.

At the first week of infection, *Schistosoma* larvae migrate from skin to the lungs, initiating immune responses and inflammation to the host [29–30]. The studies on bacterial infection, *Klebsiella pneumoniae* and *Mycobacterium tuberculosis*, showed that lung infection could affect abundances and types of fecal metabolites and microbiota [31–32]. The concept of gut-lung axis was widely discussed in many infectious and non-infectious diseases. It was assumed that gut microbiota and their metabolites play critical roles in modulation of host immunity and homeostasis [33–34]. Unfortunately, there was no evidence available for gut-lung axis in parasitic infections, but there is a possibility that schistosomula lung invasion might lead to alteration of gut microbes as observed in bacterial infections. The dysbiosis of intestinal bacteria is the common finding in schistosomiasis. Evidence has shown that the disturbance of bacterial diversity in intestine occurred with *Schistosoma* infection with, both sex [35], single sex [36], and *S*. *haematobium* that the adults do not release their eggs to intestinal lumen [37]. These studies suggested that not just parasite eggs, but the parasites themselves could cause microbiota disturbances as well. The dysbiosis provided the clue to understand our pathway analysis results at the first week of infection. Primary bile acid biosynthesis was the only pathway that was altered in intestine of *Schistosoma*-infected mice of all species at 1 week-PI (Fig 7). Bile acids are a group of steroid derivatives, which are produced in liver and further metabolized by gut microbiota into other analogs, for example, hyodeoxycholic acid, lithocholic acid [38–39]. Therefore, the perturbation of the primary bile acid biosynthesis pathway was hypothesized to be caused by changes in intestinal bacterial communities due to the migration of schistosomula.

At 2- and 4-week PI, parasites reside mainly in the liver and develop into young adults [29]. During this stage, patients still show clinical manifestations of acute schistosomiasis [8]. The pathways that we found affected in all species at 2 week-PI were primary bile acid biosynthesis, steroid biosynthesis, and biosynthesis of unsaturated fatty acids. At 4 week-PI, the affected pathways were similar to those at 2 weeks PI, with the addition of linoleic acid metabolism (Figs 6 and 7). Interestingly, the primary bile acid biosynthesis pathway was presented in all time-points, indicating the prolong alteration of this pathway. The other 4 pathways those were disturbed in these period were clearly involved with fatty acids and steroids. The explanation of these perturbation might involve gut microbiota as previously discussed. Many intestinal microbes are known to metabolize non-digestible carbohydrates into SCFA, which are a part of lipid metabolism and other vital pathways, such as energy homeostasis, inflammation, immunity [35]. Changes in SCFA and other lipid molecules might be reflected in the pathway analysis results that many lipid metabolism pathways were dysregulated. In addition to microbiota, the disturbance of these pathways might relate to the growth of parasites. The gene expression of adult worms was predominantly involved in nutritional metabolisms, when compared to schistosomula stage [40]. This finding suggested that adult *Schistosoma* spp. consume host nutrients to a greater extent than the juveniles. *Schistosoma* parasites as well as other trematodes have compromised lipid metabolism pathways [41–42]. As a result, they are unable to *de novo* synthesize their own fatty acids and sterol. They consume these lipids from the host and convert them into the forms they need. Lipids are taken into up to 50% of *Schistosoma* worms’ daily feed content [42]. With huge demand on lipid intake, changes in lipid level were constantly observed in host system of many *Schistosoma-*infected subjects, for example, blood [43–44], liver [45], and feces [22]. Thus, there is possibility that the altered lipid metabolism pathways during these periods might be caused by parasite consumption as well as dysregulation of intestinal bacterial communities.

At 8 week-PI, the affected pathways in all *Schistosoma* spp. were primary bile acid biosynthesis, steroid biosynthesis, steroid hormone biosynthesis, arachidonic acid metabolism, and GAGs degradation. The primary bile acid biosynthesis and steroid biosynthesis were similar to the previous time-points, which might be caused by the aforementioned mechanisms. Regarding steroid hormone biosynthesis and arachidonic acid metabolism, these 2 pathways are well-known for their active roles in inflammation [46–47]. During this period, eggs of parasite were produced and released to intestinal lumen, leading to inflammation of the organ. Likewise, GAGs degradation might involve in the progression of intestinal schistosomiasis as well. GAGs are the unbranched polysaccharide, composed of repeated disaccharide monomers. There are 5 types of GAGs, including hyaluronan, heparin/heparan sulfate, dermatan sulfate, keratan sulfate, and chondroitin sulfate. GAGs are usually expressed on outer membranes of the cells and extracellular matrix, which GAGs presents in both human and trematodes [48–49]. In *Schistosoma* worms, GAGs were believed to involve in anti-coagulation process [50]. In humans, GAGs are found to be associated with infection caused by many groups of pathogens, including parasites [49–50]. The impairment of intracellular GAGs degradation pathway leads to accumulation of these molecules and causes various diseases called mucopolysaccharidoses [51]. GAGs degradation can occur to GAGs on cell surface and extracellular matrix as well. In the intestinal environment, some GAGs, such as heparan sulfate proteoglycans, play key roles in maintaining tissue integrity. Once inflammation occurs, enzymes like matrix metalloproteinase increase their activity and lead to tissue damage [52–53]. The catalytic process releases degraded compounds, including GAGs and other metabolites, into the intestinal lumen, resulting in the enrichment of GAGs degradation pathways. This pathway had been identified as altered pathway from intestinal tissue of ulcerative colitis patients [54], supporting our findings on pathway analysis. In our study, we found disturbance of GAGs degradation pathway only at 8 week-PI. We hypothesize that the disturbance might occur from inflammation of intestine caused by parasite eggs, as was observed from colitis study [54]. Therefore, changes in metabolites of GAGs degradation pathway may be used as the indicator for pathological damages in the intestinal schistosomiasis.

With metabolite profiling and in-dept statistical analysis, we pinpointed 5 molecules as the potential trans-genus markers of early schistosomiasis, named 25-hydroxyvitamin D2, 1α-hydroxy-2β-(3-hydroxypropoxy) vitamin D3, Ganoderic acid Md, feature with m/z 455.3483, and feature with m/z 456.3516 (Fig 9). Peak intensity level of 25-hydroxyvitamin D2 was significantly lower than control mice at 2 week-PI only (Fig 9A). The 25-hydroxyvitamin D2 or 25-hydroxyergocalciferol is a derivative of vitamin D that is metabolized from dietary vitamin D2 from plants and mushrooms. The 25-hydroxyvitamin D2 is important for bone mineralization and regeneration. The serum total 25-hydroxyvitamin D, combined 25-hydroxyvitamin D2 and 25-hydroxyvitamin D3, is used to evaluate vitamin D levels in individuals [55]. In children, the higher levels of serum 25-hydroxyvitamin D were associated with higher levels of immunological cytokines, interleukin-6 and tumor necrosis factor [56]. In contrast, lower serum 25-hydroxyvitamin D had a significant association with higher levels of inflammation markers, C-reactive protein [57]. Regarding infectious diseases, 25-hydroxyvitamin D was proposed as a marker for detection of histoplasmosis and neonatal sepsis [58–59]. Surprisingly, Noha, *et al*. reported the decreased level of serum 25-hydroxyvitamin D in *S*. *mansoni-* and *S*. *haematobium-*infected patients, both acute and chronic forms [60]. Although their sample size was relatively small (33 patients), this finding supports our hypothesis that 25-hydroxyvitamin D2 may be an interesting target for the future development of markers for schistosomiasis. On the other hand, intensities level of 1α-hydroxy-2β-(3-hydroxypropoxy) vitamin D3 was found to be reduced at 4- and 8 week-PI (Fig 9E). The metabolite 1α-hydroxy-2β-(3-hydroxypropoxy) vitamin D3 is an analog of vitamin D3 and is relatively close to Eldecalcitol (1α,25-dihydroxy-2β-(3-hydroxypropoxy) vitamin D3), a potent drug for osteoporosis treatment [61]. Eldecalcitol can bind to vitamin D receptor and improve bone mineral density [62]. Unfortunately, 1α-hydroxy-2β-(3-hydroxypropoxy) vitamin D3 had never been proposed as biomarkers in any disease before. In fact, the association between vitamin D and schistosomiasis had been proven beforehand. The serum level of vitamin D was found to be associated with immune imbalance and progression of hepatic damages in advanced schistosomiasis patients [63–64]. Administration of a combination of praziquantel and vitamin D3 to patients with *Schistostoma haematobium* infection promoted T-helper lymphocyte type 2 reactions, which can be observed from increased specific IgE responses and percentage of eosinophil vacuolization [65]. All of these findings focused on the systemic impacts of vitamin D on schistosomiasis patients, leaving a gap in our understanding of its effects on the intestine. Biological functions of vitamin D and its analogs to intestinal environment were widely discussed, including homeostasis, gut microbiota, immunomodulation, inflammation, and pathologies [66–67]. None of them focused on effects of vitamin D to intestinal pathology of schistosomiasis, specifically to biomarker potential. Therefore, more in-depth research should be performed to understand roles of vitamin D to *Schistosoma* gut pathology, especially biomarker potential of the 2 vitamin D analogs highlighted by our study.

Ganoderic acid Md was another molecule that passed our criteria for markers in 2- and 8 week-PI groups of *Schistosoma* infection. Ganoderic acid Md is a triterpenoid compound, extracted from Chinese medicinal fungus, *Ganoderma lucidum* [68]. There are many analogs of ganoderic acid that possess various biological activities, for example, anti-tumor, hepatoprotection, neurotrophic [69]. There was only one study that mentioned ganoderic acid as a biomarker. The study of Morgan-Benita, *et al*. highlighted ganoderic acid C2 as the potential biomarker for type 2 diabetes mellitus progression [70]. In addition, there were 2 features that failed to be assigned with a metabolite: the m/z 455.3483 and the m/z 456.3516. Without metabolite assigned, it was difficult to explore their biological importance. However, there was some research that proposed unassigned features as potential markers of infectious diseases [71–72]. To leverage these unidentified features, thorough metabolite identification protocols and validation methods are needed. Although these challenging processes must be performed before point-of-care diagnosis tools can be developed, there is the possibility that these 2 features may be further developed. To strengthen the biomarkers potential of these 3 molecules, comprehensive studies are needed to explore more on their biological impacts to the parasites as well as their precise level at the early stages of the infection.

Though we successfully elucidate changes of fecal metabolites over the course of *Shcistosoma* infection and pinpointed biomarkers of the early infection, there are some limitations remains. Firstly, we used mice as the model of infection. The digestive physiology of mice and humans are not alike, leading to differences in some types of fecal metabolome. Nonetheless, one of our objectives is to identify markers of the early *Schistosoma* infection. It is impossible to screen for early infection from humans in the field setting. To answer our objective, we chose to perform our experiments in animal model and used thorough data analysis to cover this limitation. Secondly, the fecal metabolome may be influenced by ingested food. In laboratory settings, the feed of mice was controlled, which is different from the field setting where humans eat various foods every meal. We chose feces as our metabolomic samples due to it is easy and less invasive method for sample collection in the field practice. Moreover, metabolomic analysis of fecal samples provides direct evidence for alteration of digestive physiology. To address this challenge, we employed a combination of multiple methods to screen for potential biomarkers and applied strict criteria for statistical analysis to exclude features with weak potential. Thirdly, we proposed biomarkers specifically to the single infection of *Schistosoma* worms. In reality, the endemic areas of schistosomiasis usually overlap with other helminths and protozoa. There is a possibility that the fecal metabolites may be different in patients with co-infection. In addition, other types of infection were not included in this study. Therefore, we could not determine whether the proposed markers are specific to *Schistosoma* infection or if they represent a generic response to any infection. Further research is needed to explore potential of our biomarkers in patients who are infected with *Schistosoma* blood flukes and other parasites, as well as other infectious diseases. Lastly, our study had a small sample size. We applied many statistical analyses to verify our findings, however, quantification of proposed metabolites in clinical samples are needed to validate diagnostic potential of markers from this study. We proposed that future works may focus on measuring the level of potential biomarkers from our study, especially for vitamin D derivatives, on a large sample size of people in the endemic areas of many *Schistosoma* spp. The findings from this kind of study would allow a fundamental aspect of biomarker validation.

In conclusion, we performed untargeted metabolomics to elucidate biological pathway disturbances in the intestinal environment of mice infected with 3 species of *Schistosoma* parasites, *S*. *mansoni*, *S*. *japonicum*, and *S*. *mekongi*. We found that pathways in lipid metabolism were altered since the first week and throughout the infection. Once egg presented, GAGs degradation pathway was found disturbed, which this pathway might related with injuries those caused by eggs. Moreover, statistical analysis methods were applied to screen for biomarkers of early infection in all 3 species. We identified 5 potential markers, including 25-hydroxyvitamin D2, 1α-hydroxy-2β-(3-hydroxypropoxy) vitamin D3, Ganoderic acid Md, feature with m/z 455.3483, and feature with m/z 456.3516. The 5 molecules can be used for further studies aiming to discover markers of schistosomiasis at the genus level. Our findings represent an initial step towards the development of a biomarker for *Schistosoma* infection, regardless of the parasite species. The successful utilization of our findings could contribute to reducing losses and fatalities resulting from schistosomiasis.

## Materials and methods

### Ethics statement

Experiments regarding animals were performed in accordance with National Research Council of Thailand (NRCT) guidelines for the use of animals. All protocols had been priorly approved by the Faculty of Tropical Medicine–Animal Care and Use Committee (FTM-ACUC), Mahidol University (Approval number: FTM-ACUC 017/2022).

### Animal husbandry, parasitic infection, and fecal collection

Eight-week-old female ICR mice were purchased from the National Laboratory Animal Center, Mahidol University, and housed under steady environmental conditions at the Animal Care Unit, Faculty of Tropical Medicine, Mahidol University throughout the experiment. Three mice for each group were separately infected by *S*. *mansoni*, *S*. *japonicum* and *S*. *mekongi* cercaria using abdominal exposure. The infections were confirmed using modified Kato-Katz method [73]. Feces of mice were collected by placing a plastic sterile tray under the cage and picked the feces by clean forceps. Pre- (control) and post-infection at 1 week (1 week-PI), 2 weeks (2 week-PI), 4 week (4 week-PI), and 8 weeks (8 week-PI) feces of each mice were collected. The feces were immediately kept at -80°C until further analysis.

### Metabolite extraction

Metabolite extraction from feces was performed according to study of Erben, *et al* [74]. In brief, approximately 50 mg of feces was added to 200 μL of ice-cold isopropanol. The mixture was vigorously mixed for 2 minutes, then sonicated on ice for 5 minutes. The supernatant of the mixture was separated with centrifugation at 14,700 g for 15 min at 4°C, then transferred to a new tube. Supernatant was dried using speed vacuum machine (Tomy Digital Biology, Tokyo, Japan) and metabolite was resuspend with 30 μL of 2% acetonitrile in water before sending for metabolite identification.

### Metabolite identification

Metabolite identification was performed in 2 technical replications using ultra-high performance liquid chromatography (UHPLC; Agilent 1260 Quaternary pump, Agilent 1260 High Performance Autosampler and Agilent 1290 Thermostatted Column Compartment SL, Agilent Technologies, CA, USA) coupled to a quadrupole time-of-flight mass spectrometer (QTOF-MS) (TripleTOF 5600+, SCIEX, US) with DuoSpray ion source electrospray ionization (ESI). For UHPLC separation, mobile phase A, which consisted of 0.1% formic acid in water, was mixed with mobile phase B, comprising 0.1% formic acid in acetonitrile, in a 50:50 ratio. This mixture was then used for resuspending metabolite samples and subsequently transferred for injection into the liquid chromatography (LC) system. Samples were kept in a 6°C auto-sampler and 5 μL of samples was injected into the UHPLC with C18 reversed phase column (ACQUITY UPLC BEH, 2.1 × 100 mm, 1.7 μM, Waters) at the flow rate of 0.3 mL/minute at 40°C. Regarding the Q-TOF-MS system, Analyst Software version 1.7 (SCIEX) was used to acquire mass ion chromatograms and mass spectra in both positive (+ESI) and negative (-ESI) electrospray ionization modes. Data acquisition was performed with an information-dependent acquisition mode composed of a TOF-MS scan and 10 dependent product ion scans were used in the high sensitivity mode with dynamic background subtraction. The mass range of the TOF-MS scan was m/z 100–1,000 and the product ion scan was set to m/z 50−1,000. Quality control (QC) samples, created by pooling equal aliquots of each metabolite sample, were injected before, during (every 3-sample interval), and after sample analysis to evaluate system performance.

### Metabolite annotation

Metabolite annotation was performed using XCMS online platform Version 3.7.1 (https://xcmsonline.scripps.edu/landing_page.php?pgcontent=mainPage) [75]. Metabolomic raw files (.wiff and.wiff.scan) were uploaded to the XCMS server, and the ’Multi-group’ option was selected for analyzing data from the control, 1 week-PI, 2 week-PI, 4 week-PI, and 8 week-PI groups within each species separately. The processes of metabolite annotation comprised of feature extraction, alignment, annotation, and identification, which the detailed parameters were listed. The feature extraction parameters included polarity selection (either positive or negative mode) and a maximal tolerated m/z deviation of 15 ppm. Additionally, the parameters for second peak width, signal/noise threshold, and minimum difference in m/z were set to 5–20, 6, and 0.01, respectively. The alignment parameters were 5 second allowable retention time duration, 0.5 minimum fraction, and 0.015 width of overlapping m/z. The annotation parameters included 5 ppm error, 0.01 m/z absolute error, and isotopic search for the features and their adduct formations. The identification process, 74 common adducts were considered for database search with 5 ppm tolerance for database search. *Mus musculus* was chosen for biosource and METLIN database was used in the process of metabolite annotation.

### Data preprocessing and analysis

Data regarding the m/z, retention time, and intensity of all features were downloaded from XCMS online results and subsequently subjected to statistical analysis using Metaboanalyst online platform version 5.0 (https://www.metaboanalyst.ca/) [76]. Data was analyzed with “Statistic Analysis [One factor]” module. Within the module, data underwent filtering, normalization, transformation, and scaling using interquartile range, quantile normalization, cube root transformation, and range scaling, respectively. To assess the reliability of metabolite identification system, all data, including QC data, was analyzed with Principal Component Analysis (PCA). The acceptance criteria for high-quality data were that QC samples must cluster in the middle of the PCA plot [77]. Datasets that met these criteria were further analysis.

To investigate impacts of *Schistosoma* infection on fecal metabolome, fold change and significance of all features were calculated. The features whose intensities changed from the control group ≥ 2-fold, and whose *p*-values from the Mann-Whitney U test were < 0.05, were considered as significantly changed features. Furthermore, a hierarchical clustering heatmap of each *Schistosoma* species was generated to provide a comprehensive perspective of infection at different points in time, using the Euclidean distance measure and Ward clustering.

Subsequently, the multivariate model, Partial least squares-discriminant analysis (PLS-DA), was performed to investigate data separation among control samples and samples in each infection time-point. The model performance validation was assessed by Q^2^ score from leave-one-out cross-validation (LOOCV) method. In addition, variable important projection (VIP) score of PLS-DA model of all features were calculated to identify important features, the features with VIP score ≥ 1.

### Pathway analysis

The functional analysis module was used to investigate molecular pathways that were affected by the parasites. Data from all features, including m/z, mode of ionization, *p*-value, and t-score, were uploaded to create peak list profiles. Data from both positive and negative modes were combined, with a mass tolerance set to 5 ppm. The Mummichog algorithm was chosen for analyzing high through-put untargeted metabolomic data from “*Mus musculus* [KEGG]” pathway library with *p-*value cut-off of 0.05. The results were presented in scatter plots for infection of each *Schistosoma* species and infection time-points.

### Biomarker selection

The process of biomarker selection was performed using “Biomarker Analysis” module, which data was preprocessed as mentioned earlier. The Classical univariate receiver operating characteristic (ROC) curve model was chosen for analysis. The features with area under the ROC curve > 0.8, fold change ≥ 2-fold, T-test < 0.05, and VIP score from PLS-DA model ≥ 1.0 in all time-points were considered as the potential biomarkers of each *Schistosoma* spp. The overlapping potential biomarkers of *S*. *mansoni*, *S*. *japonicum*, *and S*. *mekongi* were considered as trans-genus biomarkers of intestinal schistosomiasis. In addition, trans-genus biomarkers those indicated infection before the presence of eggs, as detected by parasitological methods, were highlighted as markers of early infection.

## Supporting information

S1 Fig The PLS-DA model of metabolomic data from all species at the different time-points. A). 1 week-PI B). 2 week-PI C). 4 week-PI D). 8 week-PI. Green represents data from control group. Red represents data from infected groups. Triangle means metabolomic data from *S*. *mansoni-*infected mice. Circle means metabolomic data from *S*. *japonicum-*infected mice. Cross means metabolomic data from *S*. *mekongi-*infected mice.
(TIFF)

S1 Table Arrangement of the affected pathways according to KEGG database (with *p*-values). (XLSX)

S1 File Raw data for *S*. *mansoni* infection group. (XLSX)

S2 File Raw data for *S*. *japonicum* infection group. (XLSX)

S3 File Raw data for *S*. *mekongi* infection group. (XLSX)
