# Supplementary figures and images for: Identification of trans-genus biomarkers for early diagnosis of intestinal schistosomiasis and progression of gut pathology in a mouse model using metabolomics
Source: PLoS Negl Trop Dis. 2024 Feb 21;18(2):e0011966. doi: 10.1371/journal.pntd.0011966 (PMC10880994; doi:10.1371/journal.pntd.0011966)

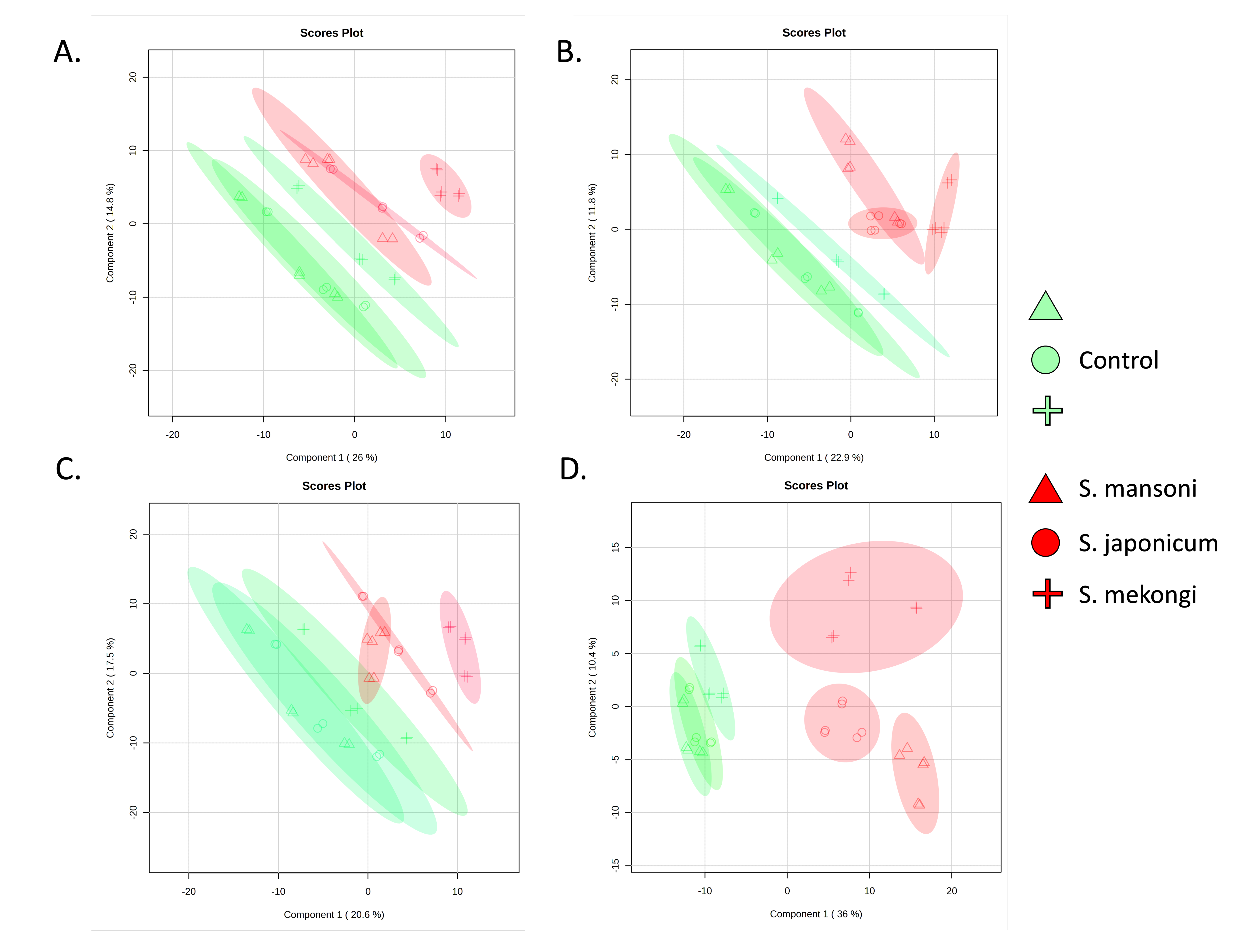

Supplement: S1 Fig — A). 1 week-PI B). 2 week-PI C). 4 week-PI D). 8 week-PI. Green represents data from control group. Red represents data from infected groups. Triangle means metabolomic data from S. mansoni-infected mice. Circle means metabolomic data from S. japonicum-infected mice. Cross means metabolomic data from S. mekongi-infected mice. (TIFF) [file pntd.0011966.s001.tiff]
